# Neural Dynamics of Olfactory Perception: Low- and High-Frequency Modulations of Local Field Potential Spectra in Mice Revealed by an Oddball Stimulus

**DOI:** 10.3389/fnins.2019.00478

**Published:** 2019-05-28

**Authors:** Jeungeun Kum, Jin Won Kim, Oliver Braubach, Jong-Gyun Ha, Hyung-Ju Cho, Chang-Hoon Kim, Hio-Been Han, Jee Hyun Choi, Joo-Heon Yoon

**Affiliations:** ^1^Brain Science Institute, Korea Institute of Science and Technology, Seoul, South Korea; ^2^Division of Bio-Medical Science & Technology, Korea Institute of Science and Technology, University of Science and Technology, Seoul, South Korea; ^3^Department of Otorhinolaryngology, Yonsei University College of Medicine, Seoul, South Korea; ^4^The Airway Mucus Institute, Yonsei University College of Medicine, Seoul, South Korea; ^5^Korea Mouse Phenotyping Center (KMPC), Seoul, South Korea; ^6^Department of Bio and Brain Engineering, Korea Advanced Institute of Science and Technology, Daejeon, South Korea

**Keywords:** oddball paradigm, local field potential, neural oscillations, olfaction, attention, anterior cingulate cortex, primary olfactory cortex, anterior olfactory

## Abstract

Recent brain connectome studies have evidenced distinct and overlapping brain regions involved in processing olfactory perception. However, neural correlates of hypo- or anosmia in olfactory disorder patients are poorly known. Furthermore, the bottom-up and top-down processing of olfactory perception have not been well-documented, resulting in difficulty in locating the disease foci of olfactory disorder patients. The primary aim of this study is to characterize the bottom-up process of the neural dynamics across peripheral and central brain regions in anesthetized mice. We particularly focused on the neural oscillations of local field potential (LFP) in olfactory epithelium (OE), olfactory blub (OB), prefrontal cortex (PFC), and hippocampus (HC) during an olfactory oddball paradigm in urethane anesthetized mice. Odorant presentations evoked neural oscillations across slow and fast frequency bands including delta (1–4 Hz), theta (6–10 Hz), beta (15–30 Hz), low gamma (30–50 Hz), and high gamma (70–100 Hz) in both peripheral and central nervous systems, and the increases were more prominent in the infrequently presented odorant. During 5 s odorant exposures, the oscillatory responses in power were persistent in OE, OB, and PFC, whereas neural oscillations of HC increased only for short time at stimulus onset. These oscillatory responses in power were insignificant in both peripheral and central regions of the ZnSO_4_-treated anosmia model. These results suggest that olfactory stimulation induce LFP oscillations both in the peripheral and central nervous systems and suggest the possibility of linkage of LFP oscillations in the brain to the oscillations in the peripheral olfactory system.

## Introduction

Olfactory dysfunction is a common condition, with a reported prevalence of 4 to 24.5% (Estrem and Renner, 1987; Murphy et al., 2002; Bramerson et al., 2004; Vennemann et al., 2008; Schubert et al., 2012). Recent epidemiological studies have shown that self-reported olfactory dysfunction is more common in men than in women (Schubert et al., 2012), tends to increase with age (Doty et al., 1984a), and is related to smoking (Frye et al., 1990). The aetiologies of olfactory dysfunction are various (Doty, 2006), but are often classified as follows: (1) conductive impairment from the blockage of nasal airflow (e.g., rhinosinusitis and polyposis) (Deems et al., 1991), which happens in most patients with hyposmia or anosmia; and (2) sensorineural impairment from damage. Sensorineural impairment can be divided into two types. The first type is peripheral olfactory dysfunction, which occurs following damage to the OE due to upper respiratory infections, among other causes (Deems et al., 1991; Suzuki et al., 2007). The second type is central olfactory dysfunction, which occurs as a result of neurodegenerative diseases (e.g., Parkinson’s disease, dementia with Lewy bodies, frontotemporal dementia, and Alzheimer’s disease) (Devanand et al., 2000; Muller et al., 2002), neurological and neuropsychiatric disorders (e.g., Kallmann syndrome (Lieblich et al., 1982), epilepsy (Eskenazi et al., 1986), and schizophrenia (Turetsky et al., 2000), or head trauma (Zusho, 1982). Recent brain connectome studies have shown that the projections from the OB to the central brain regions such as piriform cortex, HC, amygdala, and hypothalamic nuclei^1^. However, the dynamics of how the bottom-up signals of odorants propagate to the central brain regions are poorly known, limiting the diagnostic evaluation of hypo- or anosmia in olfactory disorder patients.

Objective assessments of olfactory perception are critical for the diagnosis and treatment of olfactory dysfunction. As such, numerous odor identification tests have been developed for examining olfactory disorders. Among the most commonly used tests are the University of Pennsylvania Smell Identification Test (Doty et al., 1984b), Cross-Cultural Smell Identification Test (Doty et al., 1996), European Test of Olfactory Capabilities (Thomas-Danguin et al., 2003), and Sniffin Sticks test (Hummel et al., 1997). These test batteries consist of perception threshold, odor discrimination, and identification tests, which are mainly based on an individual’s subjective responses. Given their reliance upon subjective responses, these tests are often accompanied by more objective olfactometric analyses (e.g., dose-response relationship or exposure-response relationship analyses). However, the use of olfactometric analyses is limited, as such analyses can be difficult to incorporate into routine clinical evaluations. Another objective assessment is electro-olfactography (EOG), which measures peripheral olfactory function at the level of olfactory receptors within the nasal cavity. Various researchers have used EOG to extensively characterize the responses in both humans and animals (Shibuya, 1964; Okano and Takagi, 1974; Scott and Scott-Johnson, 2002), but this electrophysiological approach can only be used to evaluate the peripheral nervous system. Therefore, other methods that can examine olfactory function in both the central and peripheral nervous systems are needed.

Olfactory deficits as a consequence of central nervous system disease have been studied with non-invasive brain mapping techniques such as functional magnetic resonance imaging, positron emission tomography, magnetoencephalography, and electroencephalography (EEG) (Hawkes et al., 1997; Boesveldt et al., 2009; Baba et al., 2012; Schriever et al., 2017; Versace et al., 2017). Functional magnetic resonance imaging and positron emission tomography are increasingly being used to elucidate the specific areas of olfactory deterioration both in healthy individuals and in those with neurological and neurodegenerative diseases (Wang et al., 2005; Hummel et al., 2010; Meles et al., 2017). Several studies have revealed that olfactory deficits are not only indicated by changes in specific olfactory areas but also by changes in the functional connectivity of the olfactory network (Kollndorfer et al., 2015; Reichert et al., 2018). Recently, the neural correlates of olfactory deficits in neurodegenerative diseases have been identified, such as absent olfactory event-related potentials or decreased alpha 1 (8–10 Hz) power of the olfactory responses recorded via magnetoencephalography in patients with Parkinson’s disease (Boesveldt et al., 2009; Versace et al., 2017) and weaker blood oxygen level-dependent signals in the primary olfactory cortices and insular cortices of patients in the early stages of Alzheimer’s disease (Wang et al., 2010). Another study was able to reliably distinguish patients with olfactory impairment from healthy individuals by using time-frequency analysis of EEG (Schriever et al., 2017). However, there is inherently spatial and temporal insensitivity in many neuroimaging studies (Gottfried and Zald, 2005), as well as other limitations such as absent or unsatisfactory responses in olfactory evoked potential studies (Hawkes, 2006) and the inability to understand whether the olfactory deficit emanates from a peripheral nervous system disorder or a neurodegenerative central nervous system disorder. To obtain a diagnostic profile of deficits in olfaction, it will be critical to characterize the neurodynamic responses to odor exposure in the olfactory regions of both the nasal cavity and the brain.

Our aim in this study was to characterize odor-evoked neural oscillation patterns via invasively recording local field potentials (LFPs) simultaneously from both the peripheral (OE) and central (OB, PFC, and HC) nervous systems using a passive oddball paradigm. The brain areas of interest were chosen based on their relevance to olfactory consciousness or processing, as well as their detectability in human EEG. For example, beta and gamma oscillations in the PFC are known to reflect olfactory consciousness (Mori et al., 2013) and theta oscillations in the HC are known to be driven by olfactory input (Kay et al., 2009). In our study, we presented two different odorants under the oddball paradigm and characterized the olfactory evoked oscillations for both repetitive and rare stimuli in multiple sites of brains. In order to exclude the neuromodulation of olfaction via top-down regulation, we applied urethane anesthesia and delivered a precise amount of odorant via computerized olfactometer. A time-frequency analysis of slow and fast neural oscillations was conducted to identify the possible occurrence of long-term habituation on control mice and to compare the responses with the ones obtained from anosmia mouse model with epithelial injury.

## Materials and Methods

### Animals

All surgical and experimental procedures were followed by Korean Animal and Plant Quarantine Agency Publication No. 12512, partial amendment 2014, conforming to NIH guidelines (NIH Publication No. 86–23, revised 1985). All the procedures were approved by the Institutional Animal Care and Use Committee of the Korean Institute of Science and Technology (AP-2014L7002). Ten mice (C57BL/6N, 8–12 weeks old, body weight: 22–28 g) were used in this study. The animals were housed in a colony maintained on a 12-hour light/dark cycle at 22°C with 55% humidity and allowed *ad libitum* access to food and water. The animals were born and raised in this specific pathogen-free environment.

### Surgery and LFP Recordings

To implant the electrode for the LFP recordings, we first anesthetized the mice by administering urethane (1.5 g/kg) intraperitoneally, and then placed and fixed the mice in a stereotaxic apparatus (Model 902; David Kopf Instruments, Tujunga, CA, United States). All LFP recordings were performed with sterile bipolar Teflon-coated tungsten electrodes (Model No. 796000, A-M Systems, Sequim, WA, United States). The electrode impedance was 100 – 300 kΩ when measured with an impedance tester (test frequency at 1 kHz, nanoZ, Plexon, Inc., Dallas, TX, United States). To record OE, the electrode was placed in or near the left or right olfactory mucosa (just rostral to the nasofrontal suture, 7.0 mm anterior and 0.3 mm lateral to the bregma point, 0.5 mm below the skull surface). In the brain, three electrodes were implanted in OB (anteroposterior: 4.8 mm, mediolateral: 1.2 mm, dorsoventral: −1.1 mm from bregma), PFC (anteroposterior: 0.5 mm, mediolateral: 1.54 mm, dorsoventral: −2.7 mm from bregma), and HC (anteroposterior: −2.0 mm, mediolateral: 1.5 mm, dorsoventral: −1.4 mm from bregma). For the ground electrode, a sterilized micro screw was implanted on the interparietal bone. All stereotaxic coordinates of the electrodes were in accordance with the mouse brain (Paxinos et al., 2001).

After the surgery, we calibrated the density of odorant and ensured a quick and complete vacuuming of odorant with a photo-iodine detector (miniPID 200B; Aurora scientific, Aurora, ON, Canada). Then the mice were moved to a custom-made mouse restrainer with their heads fixed in front of the olfactometer. The nose tip of the animal was placed in front of the olfactometer outlet with a 1-cm gap. We recorded LFPs during the oddball task with a Cerebus amplifier (Blackrock Microsystems, Salt Lake City, UT, United States). All signals were digitized with a 2-kHz sampling rate and bandpass filtered from 0.3 to 500 Hz.

### Odor Oddball Paradigm

**FIGURE 1:**
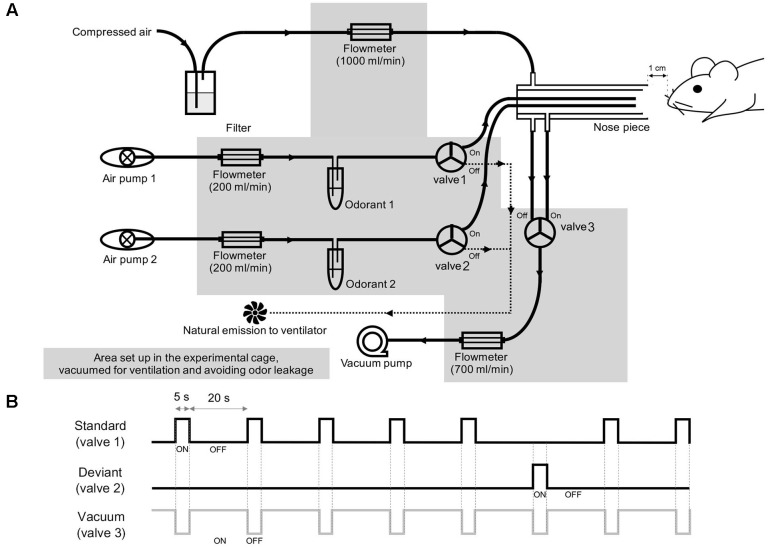
Experimental setup. **(A)** Schematic diagrams of the olfactometer that was used to deliver the two odor stimuli. **(B)** Schematic overview of the two-odor oddball paradigm. The standard, deviant, and vacuum channels were paired with separate solenoid valves, which are numbered in **(A)**.

The olfactometer was designed to provide a constant flow of air at the tube outlet and quick vacuuming of odors (Figure 1A). Each odor was diluted from a saturated vapor with filtered air and vaporized by pumped air via an air pump (Active Aqua Air Pump (two outlets, 3 W); Hydrofarm, Inc., Petaluma, CA, United States). The streaming of odors was blocked or opened by a solenoid valve (HTV 0301-3; KCCPR, Seoul, South Korea), which was controlled by custom-built MATLAB software (The MathWorks, Inc., Natick, MA, United States). When the solenoid valve was open, the odor was constantly injected into the animal’s naris, whereas when the solenoid valve was closed, the air was naturally emitted from the ventilator. For constant airflow, the nose piece was designed to have two layers, an outer layer for constant delivery of filtered air and an inner layer for the delivery of odors. Within the inner layer, two tubes were aligned to deliver the odors. To avoid cross-contamination, a tube was connected from the inner layer to a vacuum pump (Vario 18; Medela, Baar, Switzerland), which was coupled to the solenoid valve and opened to remove the odors.

For the olfactory oddball paradigm, methyl salicylate (>99% purity, mineral oil solution, ratio of odor to solvent was 3:1; Sigma Aldrich, Inc., St. Louis, MO, United States) and ethyl acetate (>99.5% purity, distilled water solution, odor to solvent ratio was 1:1 Sigma Aldrich, Inc., St. Louis, MO, United States) were used at 10% of saturated vapor for all odor experiments. The rising and falling times of the olfactometer were approximately 100 and 130 ms, respectively, with no statistical difference between the two odors (Welch’s *t*-test, *p* > 0.05). For odor delivery, a constant flow of filtered air (1 L/min) was delivered through the outer layer of the nose piece, while odor stimuli were diluted by pumped air (200 mL/min). The solenoid valve connected to the vacuum pump was opened at the end of the stimulation (1.5 L/min) to directly remove odor stimuli. In this study, we used methyl salicylate and ethyl acetate as odorants. To avoid the odorant-type effect, we applied methyl salicylate and ethyl acetate as standard and deviant stimuli, respectively, to half of the mice (3 control and 2 ZnSO_4_-treated mice) and applied ethyl acetate and methyl salicylate as standard and deviant stimuli, respectively, to the other half of the mice (2 control and 3 ZnSO_4_-treated mice). The standard and deviant odors were randomly shuffled with a 5:1 ratio, as depicted in Figure 1B. The stimulation period was 5 s and the inter-stimulus interval was 20 s. The rising and falling phases were measured by a photo-iodine detector (miniPID 200B; Aurora scientific, Aurora, ON, Canada). A session was composed of 84 stimuli (70 standard stimuli and 14 deviant stimuli) lasting 35 min. For each mouse, we performed three or four experimental sessions, with an inter-session interval of >30 min. After the experiment, the animals were sacrificed for histological confirmation of the electrode tip positions.

### Data Analysis

All LFP signals were normalized by the average power of the signals by MATLAB software (The MathWorks, Inc., Natick, MA, United States) in the 150–200 Hz frequency range from the 5-min baseline period to match the interface impedance for all electrodes.

For the single-trial LFP display, a Butterworth filter was used for all bands of interest including the delta (δ, 1–4 Hz), theta (θ, 6–10 Hz), beta (β, 15–30 Hz), low gamma (low γ, 30–50 Hz), and high gamma (high γ, 70–100 Hz) bands. For the *z*-score power analysis, a short-time Fourier transform was used to evaluate the change in the normalized power in 1-s time windows at a time resolution of 100 ms and frequency resolution of 0.5 Hz [*z*-score = (*P*_stim_ – *P*_base_)/*P*_base_]. An epoch was extracted for every stimulus from 5 s before the onset to 15 s after the offset, and the epochs contaminated by noise were excluded. Deviant stimulation trials and standard stimulation trials before the deviant trials were used in the difference analysis. For the power spectra of LFPs, the baseline and stimulus period powers of the standard stimulation were divided by the total power of the baseline data (5 s) for each frequency. To measure within-trial decreases in oscillatory power, six control mice standard trials were aligned by trial order and the *z*-score power of each trial was averaged. In this study, we performed a Chi-square test for normality and then applied Welch’s *t*-tests for normally distributed data and non-parametric Wilcoxon rank-sum tests for non-normally distributed data.

### Anosmia Model With ZnSO_4_-Treated OE Injury

Bilateral intranasal perfusion with 20 μL of 10% ZnSO_4_ was accomplished to destroy olfactory sensory neurons with a 0.3-M chloral hydrate solution (5 mg/g body weight) for four mice. The ZnSO_4_ treatment is known to destroy the mature olfactory neuroreceptors without affecting progenitor cells, thus allowing subsequent neurogenesis processes to occur (Cancalon, 1982). The destruction of the OE was confirmed histologically using hematoxylin and eosin staining and anti-olfactory marker protein (anti-OMP antibody, Osenses Pty Ltd., Keswick, Australia) immunostaining after the experiments. The detailed procedures and treatment effects are described in our previous study (Cho et al., 2018). Six mice that received bilateral intranasal perfusion with 20 μL of a 0.3-M chloral hydrate solution served as the control group.

## Results

### Olfactory Responses in Control Mice

**FIGURE 2:**
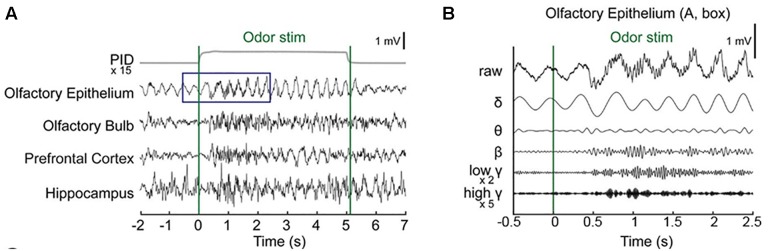
Simultaneous recording of local field potentials (LFPs) during odor stimulation. **(A)** Example of stimulus-locked LFPs and the odorant concentration. Single-trial LFPs are displayed after being bandpass filtered with a cut-off frequency from 1 to 100 Hz. Averaged photo ionization detector (PID) signals averaged over 70 trials (one session) are depicted for the odorant concentration. The vertical lines indicate the onset and offset moments of odor release. Methyl salicylate was released in this trial. The rising time of the tested odorants in this experiment was ∼100 ms. **(B)** Examples of the frequency components of the LFPs in the early responses (∼2.5 s) of the olfactory epithelium. Specifically, the δ (1–4 Hz), θ (6–10 Hz), β (15–30 Hz), low γ (30–50 Hz), and high γ (70–100 Hz) bands.

Baseline OE and OB activity consisted of high-amplitude, regularly fluctuating slow oscillations (1–4 Hz) and low-amplitude, irregular fast waves. In response to odor release, rhythmical changes were observed in a prominent way in all recorded areas (Figure 2A,B and Supplementary Figure S1). In the OE, clearδ oscillations were elicited at odor exposure and were accompanied by fast responses. Fast rhythms occurred during odor exposure (Figure 2B).

**FIGURE 3:**
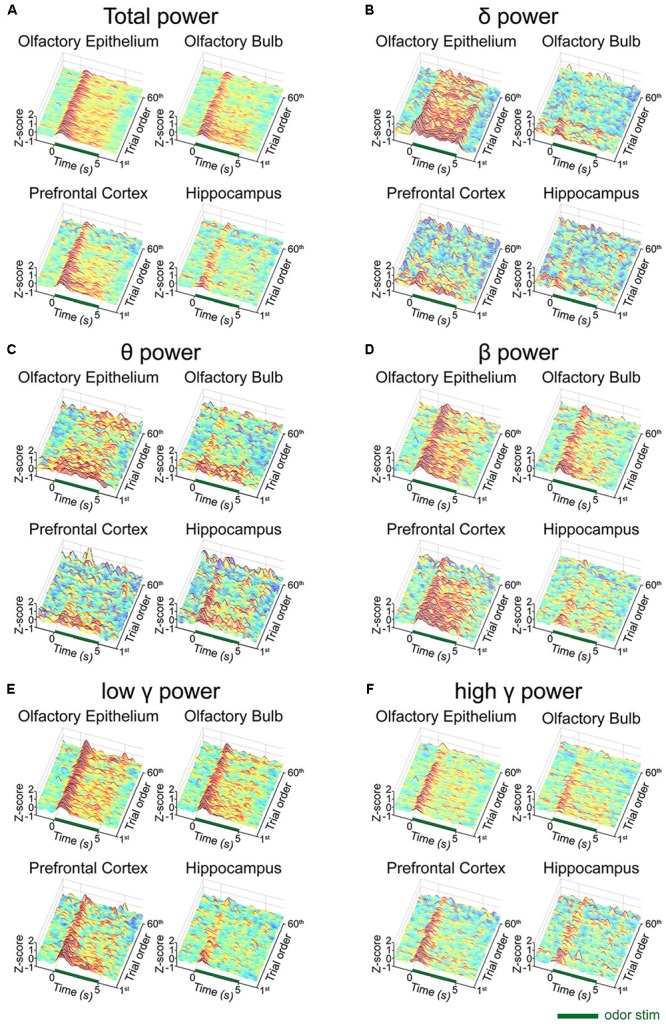
Desensitization patterns of the local field potential (LFP) signals at each channel. The mean *z*-scores of each trial of the standard stimulation condition are aligned by trial number. The *x*-axis indicates the time course of each trial from the 2-second baseline period to the post-stimulation period. The y-axis indicates the first 60 standard trials during each session. **(A)** Mean *z*-score of the total power (1–100 Hz). **(B)** Mean *z*-score of δ (1–4 Hz). **(C)** Mean *z*-score of θ (6–10 Hz). **(D)** Mean *z*-score of β (15–30 Hz). **(E)** Mean *z*-score of low γ (30–50 Hz). **(F)** Mean *z*-score of high γ (70–100 Hz).

We next investigated whether any desensitization patterns appeared over the exposure time and across the trials by plotting the time traces of the oscillatory powers across the trials (Figure 3). In order to ensure that all parameters were comparable, we calculated the *z*-scores by subtracting the baseline power from the power of each frequency band and then dividing it by the baseline power. In the OB, PFC, and HC, fast rhythms were more eminent than were slow rhythms. The LFP power spectral density was increased by odor release across the frequency bands included in the δ, θ, β, low γ, and high γ bands. Generally, the power shows that the initial strong responses were attenuated over time but the patterns were different among the regions. The temporal profiles of powers show that the elevated powers persisted in OE, OB, and PFC during the stimulation period, while the change ceased early in HC (Supplementary Figure S2).

To investigate the trial effect, we performed a linear regression analysis of power across the trials. We performed the analysis for early (0–2 s) and late (3–5 s) periods, separately, to observe when the repetition of trials has an influence. Generally, the influences of trial repetition were similar for the early vs. late periods: δ power of OE, θ power of OE and OB, low γ power of PFC decreased significantly as trial repeated (*p* < 0.05, one-sided *t*-test). On the other hand, β powers in OE and PFC, and high γ power of PFC decreased significantly only during early period and δ power of PFC decreased significantly only during late period (*p* < 0.05, one-sided *t*-test) as the trial was repeated. All results of linear regression and test of non-zero slope were depicted in Supplementary Figure S3.

**FIGURE 4:**
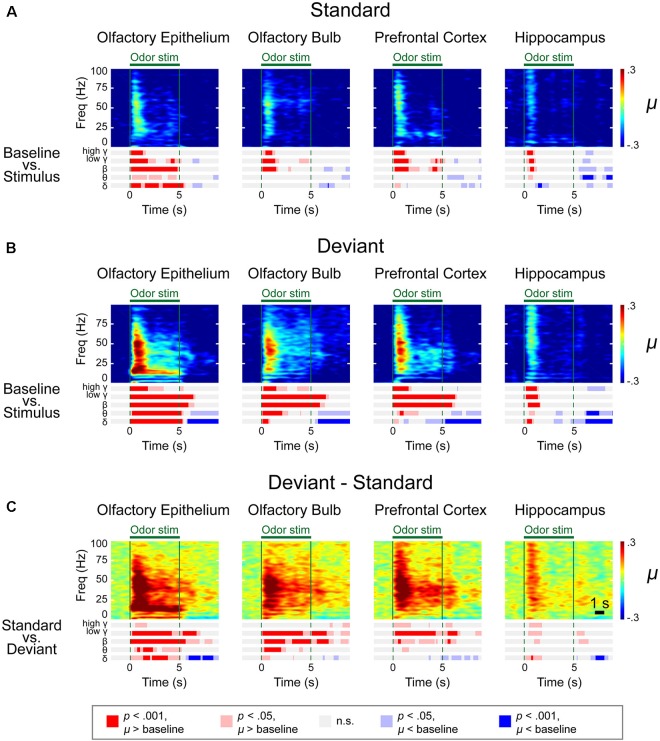
Spectrograms of the μ values of the lognormal fit of the *z*-score power from two different stimulation conditions in control mice. Methyl salicylate was used for the standard stimulation condition and ethyl acetate was used for the deviant stimulation condition or vice versa. The bars underneath the spectrograms indicate the statistical significance at each frequency band (Wilcoxon rank-sum tests). The red or blue bar indicates *p* < 0.001 and pink or sky blue bars indicate 0.001 ≤ *p* < 0.05. δ (1–4 Hz), θ (6–10 Hz), β (15–30 Hz), low γ (30–50 Hz), and high γ (70–100 Hz). **(A)** Standard trial μ value spectrogram. **(B)** Deviant trial μ value spectrogram. **(C)** Deviant – standard trial μ value differential spectrogram.

We observed distinctive response patterns for different frequencies by comparing the power values during stimulation to baseline powers. The significantly changed time periods were presented in the bar graphs under the spectrograms (Figure 4). For the δ power, the OE showed the most dramatically increased response compared to baseline during all trials, while the δ power of the other examined regions were weaker than that in the OE. High γ was increased when compared to baseline in all regions. The θ power of the stimulation period was reduced in the three central nervous system regions during repeated trials, while the θ power in the OE remained high during repeated trials. The HC showed brief changes at the odor onset in all trials.

### Responses to Deviant Odors During the Oddball Paradigm

A time-frequency analysis was conducted on the spectrogram of each LFP using a frequency bin width of 0.5 Hz and a temporal bin width of 1 s with a moving window of 100 ms. We first obtained the averaged spectrograms of the baseline-adjusted *z*-score for two different types of stimulation (methyl salicylate as the standard odor stimulus and ethyl acetate as the deviant odor stimulus or vice versa), as presented in Supplementary Figures S4, S5. We also calculated the differential spectrograms by averaging the differential spectrograms for the deviant stimulus compared to the previous standard stimulus. Given that the power decreases as the frequency increases with the power law, we used the *z*-scores (ratio of the stimulus power subtracted by stimulus power to baseline power) to draw the spectrograms.

To merge the spectrograms from two different stimulation conditions, we fitted the *z*-score distribution of each data point from a 500-ms window timescale with a lognormal distribution and collected the μ value, an average of the logarithmic power in the 500-ms window (Figure 4). A rapid response was observed after odor stimulation. To identify the significant differences in the responses and frequencies compared to baseline (Figure 4A,B and Supplementary Figures S4–S6) or between the standard and deviant stimuli (Figure 4C,D and Supplementary Figures S4–S6), we performed a Wilcoxon rank-sum test with a significance level of 0.05. The bar graphs under the spectrograms indicate the significant responses at each rhythm. We observed two types of responses: (i) persistent responses, where the significant increase of oscillatory power was maintained throughout odor exposure and (ii) transient responses, where the oscillatory power significantly increased briefly only at stimulus onset.

In the OE, OB, and PFC, significant responses appeared at ∼100 ms after odor stimulation for both the standard and deviant stimuli. The power of these rapid responses was significantly stronger for the deviant stimuli than for the standard stimuli in all examined regions, indicating that the mice responded to the oddball stimulation paradigm. On the other hand, this response appeared later in the HC than it did in the other measured regions, with an approximate delay of 100–200 ms. Additionally, in the HC, the significant responses were short-lived for all frequency bands.

The response patterns to the standard and deviant stimuli were different in all channels. For example, persistently elevated responses of β and low γ bands were observed with respect to the deviant stimuli but not with respect to the standard stimuli. In the case of the OE, the persistent responses of all oscillations were observed but the significance was stronger for the deviant stimuli compared to the standard stimuli.

While persistent responses of some frequency bands were observed in OE, OB, and PFC, all responses in the HC were transient for all frequency bands. When we compared the HC responses during standard versus deviant stimuli, θ power did not show any statistically significantly different power in HC. Also, in HC, the undershooting of power was more eminent particularly in δ and θ frequencies with respect to deviant stimuli. For standard stimuli, reduction of the δ and θ oscillations were observed after the cessation of odor stimulation in the OE and OB and during the late period of odor stimulation in the PFC and HC (Figure 4A). For deviant stimuli, steady and longer desynchronization of the δ and θ oscillations were observed in each area after the cessation of odor stimulation or during the late period of stimulation (Figure 4B). The differential spectrogram revealed that the desynchronization of the δ oscillations was greater for the deviant than for the standard stimulation condition (Figure 4C). Averaged spectrograms of the two different conditions are shown in Supplementary Figures S4, S5. Both conditions showed immediate responses after odor stimulation, while late-period responses showed different patterns.

The differential spectrogram (Figure 4C) revealed that the power of the δ to high γ rhythms increased in the OE during stimulation. As the pink and red bar indicates the significance, in the OB, the power of the δ to low γ rhythms increased during the stimulation period compared to baseline. In the PFC, the β to high γ power increased during odor stimulation compared to baseline. The β and low γ in the OE, OB, and PFC showed persistent responses. Unlike the other central nervous system regions, the HC demonstrated transient responses to deviant stimuli in all oscillatory powers.

### Significantly Reduced Oscillations in ZnSO_4_-Treated Mice

**FIGURE 5:**
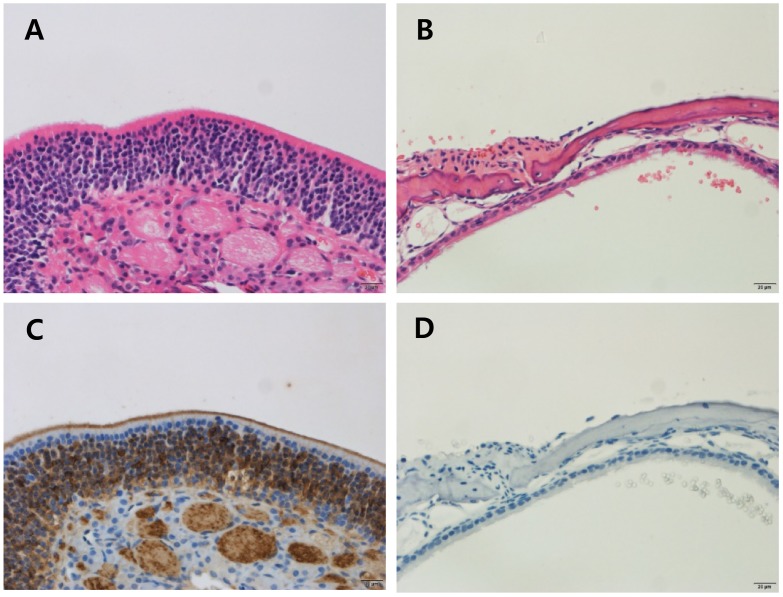
Histology of the olfactory epithelium in control and ZnSO_4_-treated mice. Olfactory epithelial tissues were stained using hematoxylin and eosin (H & E) to determine the pathology **(A,B)** and immunostained with the anti-olfactory marker protein (OMP) antibody to identify mature olfactory response neurons **(C,D)**. **(A)** Olfactory epithelial tissue from control mice showing an intact olfactory epithelium, which is composed of olfactory sensory neurons, supporting cells, and basal cells. **(B)** Olfactory epithelial tissue from ZnSO_4_-treated mice showing an olfactory epithelium that is disrupted and detached from the turbinate bone. **(C)** Olfactory epithelial tissue from control mice showing strong expression of OMP. **(D)** Olfactory epithelial tissue from ZnSO_4_-treated mice showing the absence of OMP-stained cells.

The odor evoked LFPs were investigated in a peripheral anosmia model. We produced peripheral anosmia by injecting ZnSO_4_ directly into both sides of the OE. The hematoxylin and eosin stained and anti-olfactory marker protein immunostained images revealed disruption of the epithelial tissue as well as the absence of mature olfactory response neurons (Figure 5).

We first analyzed the pre-stimulus power to investigate whether the damage of OE has altered the baseline rhythmical fluctuations of OE and brain regions. We have found that not only OE but also OB, PFC, and HC presented altered baseline powers (Supplementary Figure S7). Briefly, δ power significantly decreased in OE and OB of ZnSO_4_–treated mice compared to the control mice. On the other hand, θ power significantly increased in all recorded area of ZnSO_4_–treated mice. Significant increase of β power was observed in OE, OB, and HC in ZnSO_4_–treated mice.

**FIGURE 6:**
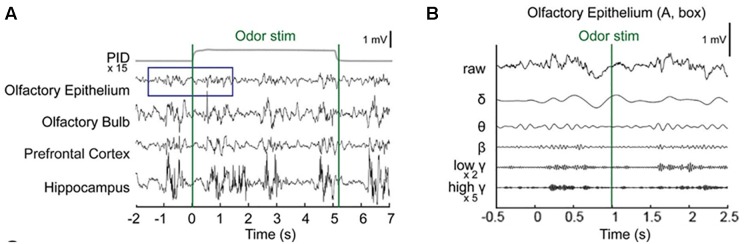
Stimulus-locked local field potentials (LFPs) in ZnSO_4_-treated mice. **(A)** Examples of stimulus-locked LFPs from ZnSO_4_-treated mice do not show differences from before to after odor stimulation in the raw time traces of single-trial LFPs. Averaged photo ionization detector (PID) signals averaged over 70 trials (one session) are depicted for the odorant concentration. The vertical lines indicate the onset and offset moments of odor release. Methyl salicylate was released in this trial. The rising time of the tested odorants in this experiment was ∼100 ms. **(B)** Examples of the frequency components of the LFPs from before stimulation to the early responses (–1.5∼1.5 s) in the olfactory epithelium. Specifically, the δ (1–4 Hz), θ (6–10 Hz), β (15–30 Hz), low γ (30–50 Hz), and high γ (70–100 Hz) bands.

In order to investigate the evoked responses, we performed the spectral analysis with respect to standard and deviant odorants. Compared to control mice, which showed an immediate increase in power in all frequency bands, the LFPs of ZnSO_4_-treated mice did not show any prominent differences during odor exposure compared to baseline (Figure 6A,B). In the OE, the amplitudes of filtered oscillations from δ to high γ were the same before and after odor exposure (Figure 6B), which contrasted with the filtered time traces of control mice.

**FIGURE 7:**
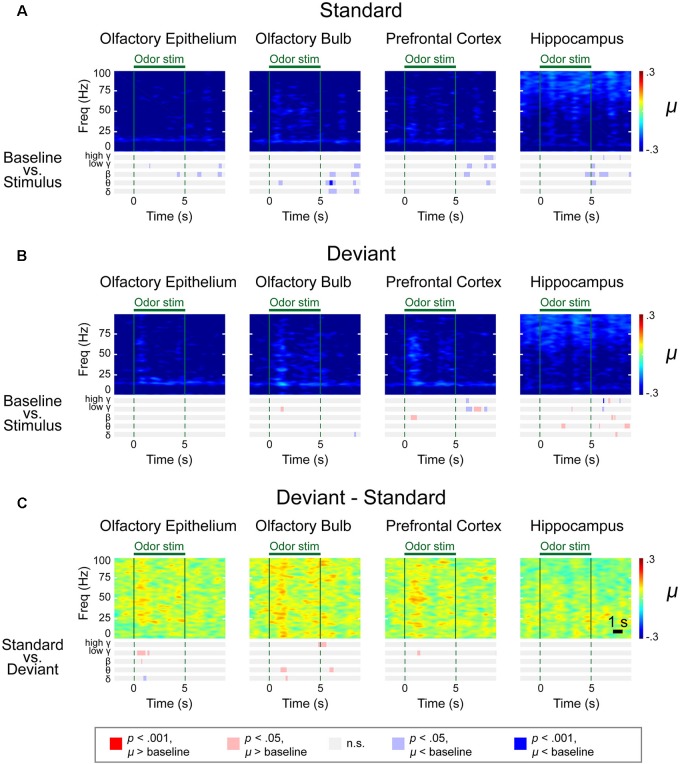
Spectrograms of the μ values of the lognormal fit of the *z*-score power from two different stimulation conditions in ZnSO_4_-treated mice. Methyl salicylate was used for the standard stimulation condition and ethyl acetate was used for the deviant stimulation condition or vice versa. The bars underneath the spectrograms indicate the statistical significance at each frequency band (Wilcoxon rank-sum tests). The red or blue bar indicates *p* < 0.001 and pink or sky blue bars indicate 0.001 ≤*p* < 0.05. δ (1–4 Hz), θ (6–10 Hz), β (15–30 Hz), low γ (30–50 Hz), and high γ (70–100 Hz). **(A)** Standard trial μ value spectrogram. **(B)** Deviant trial μ value spectrogram. **(C)** Deviant – standard trial μ value differential spectrogram.

Our time-frequency analysis revealed impaired olfactory responses in all recorded regions of ZnSO_4_-treated mice and a lack of significant responses in all regions during exposure to standard stimuli, although transient responses were observed in the OB and PFC during exposure to deviant stimuli (Figure 7). In the OE, no statistical differences were observed for any of the frequencies from θ to high γ compared to baseline in the ZnSO_4_-treated mice. In the OB, the low β response of ZnSO_4_-treated mice was reduced compared to that of control mice. In the PFC and HC, statistical differences in the θ to high γ frequencies between the baseline and stimulation periods were reduced in ZnSO_4_-treated mice when compared to the differences in control mice.

The averaged power spectrogram of the two groups, in which different standard and deviant stimuli were used on ZnSO_4_-treated mice, as previously described, showed transient responses for both the standard and deviant conditions in the OE, OB, and PFC, but the response patterns were slightly different (Supplementary Figures S8–S10).

## Discussion

In this study, we simultaneously measured LFPs from the main OE, a peripheral area that contains olfactory sensory neurons, the OB, the direct receiver of signals from olfactory sensory neurons, and other higher-order regions, including the PFC and HC, using an olfactory oddball paradigm in both healthy control and ZnSO_4_-treated anosmic mice. With this measurement, we observed the absence of odor responses in ZnSO_4_-treated mice compared to healthy control in both peripheral and central regions.

Concerning the emergence of peripheral nervous system neural oscillations along with central nervous system oscillations during odor stimulation, our results showed that OE oscillations from δ to high γ emerged during odor stimulation. These findings are in line with previous studies, including one study that simultaneously observed high frequency oscillations at the olfactory mucosa and gamma waves in the rat piriform cortex (Vanderwolf, 2000), as well as EOG studies that observed 10–60 Hz oscillations in various vertebrates, from fish, amphibians, and reptiles to mammals (Hamilton and Kauer, 1989; Suzuki et al., 2004; Lapid and Hummel, 2013; Mori et al., 2013). The persistent responses of the OE are consistent with a previous *in vivo* catfish study that identified olfactory organ oscillations referred to peripheral waves (Parker et al., 2000). While many of these studies focused on specific oscillatory bands, we observed that δ to high γ rhythms emerged simultaneously during odor stimulation in the OE.

Slow oscillations with a frequency lower than 5 Hz have been known to be reported in multiple brain regions, including OB, primary olfactory cortices, frontal cortex, and HC during sleep and anesthetic states (Kay et al., 2009; Ito et al., 2014; Yanovsky et al., 2014; Chi et al., 2016). These rhythms are called slow oscillations (Yanovsky et al., 2014) or respiratory rhythms (Kocsis et al., 2018). Recently, there were discussions on whether respiration-related slow oscillations reflect internal processing or are simply entrained by respiratory inputs (Lockmann et al., 2016). Observations with increased coherence of PFC and HC oscillations on 2–5 Hz during thalamic nucleus reticularis (nRE) stimulation indicate that respiratory rhythm could be influenced by internal processing (Kocsis et al., 2018). On the other hand, HC slow rhythms, which is coherent with respiratory-related oscillations, could be generated independently from hippocampal theta oscillations (Chi et al., 2016; Lockmann et al., 2016).

Fast oscillations have been reported in both awake and anesthetized mice. For example, β oscillations have been observed during exploration or odorant discrimination or memory tests (Kay et al., 2009; Kay, 2014; Fourcaud-Trocme et al., 2014) as well as under urethane anesthesia with olfactory stimulation (Neville and Haberly, 2003; Fourcaud-Trocme et al., 2014). For the generation of β oscillations, olfactory cortices, such as pyriform cortex are suggested as taking roles for influencing and cooperating with OB (Kay et al., 2009; Martin and Ravel, 2014). Likewise, γ oscillations are also observed on urethane anesthetized rat during odor stimulation (Neville and Haberly, 2003; Fourcaud-Trocme et al., 2014). Compared to β oscillations, odor-evoked γ oscillations are known as local oscillations of OB (Kay et al., 2009), with multiple studies indicating that the synaptic connection of OB mitral cell and granule cell underlies the generation of OB γ oscillation (Lepousez et al., 2010; Mori et al., 2013; Fukunaga et al., 2014). γ oscillations are delivered with the respiratory cycle and are involved in fine discrimination of odor (Beshel et al., 2007; Kay et al., 2009; Kay, 2014).

The present study also showed that these oscillations emerge from both peripheral and central nervous system regions simultaneously during odor perception. Dorries and Kauer (2000) speculated that the *in vivo* olfactory epithelial oscillations that they observed in the salamander may be linked to oscillations observed in the OB. Considering that the OB oscillations modulate the responses in the primary olfactory cortices and PFC (Kay et al., 2009), OE oscillations might be intermediated by the OB. In a previous study, the repetition attenuation observed in the OB is correlated with a reduction in the mitral-tufted cell firing rate during repetitive odor exposures, while the repetition attenuation observed in the OE is correlated with the olfactory receptor neuron firing rate, although the repetition attenuation in the OE is less than that in the OB (Philpot et al., 1997).

Despite these findings, the underlying function of the strengthened responses that were observed herein during exposure to deviant stimuli under anesthesia, in both the peripheral and central nervous systems, remains unclear. Clarifying this is especially important since olfactory sensory neurons in the OE are a bundle of independent sensors and are known to be unidirectional to the OB. Currently, there are two possible explanations for the strengthened responses. The first explanation is that the responses to standard stimuli are saturated while the responses to deviant stimuli are not saturated, and the second explanation is related to the perception-based modulation observed in the passive oddball response during unconscious situations (Silverstein et al., 2015). Animals anesthetized with urethane lose less sensory perception than animals anesthetized with other anesthetics do, depending on the pharmacological profile of the urethane, which has been described to consist of modest potentiation of gamma-aminobutyric acid-ergic and glycinergic transmission with modest depression of α-amino-3-hydroxy-5-methyl-4-isoxazolepropionic acid and *N*-methyl-D-aspartate receptors, which is similar to the function in awake animals (Hara and Harris, 2002; Neville and Haberly, 2003). This characteristic might contribute to the processing of sensory signals during an unconscious state. However, in both explanations, the mechanisms of the difference between the responses to standard and deviant stimuli in the OE are unknown, while the oddball response is known to influence higher-order regions (Silverstein et al., 2015). Further studies are needed to clarify the mechanism of oddball response on anesthetized animals.

In the present study, we selectively destroyed the mature olfactory neuroreceptors leaving progenitor cells intact with a treatment of ZnSO_4_ for test model (Cancalon, 1982) rather than creating lesions of the OE. The ultimate effect of ZnSO_4_ has been known to prohibit the axonal transportation to cells in the inner layers of the OB (McBride et al., 2003) and deactivate the glomerular layer of the OB (Cho et al., 2018). The destruction of OE has been reported in tetrodotoxin-treated model (Fletcher and Wilson, 2003), of which all types of cells were inhibited. Here, we observed similar effects in ZnSO4 treated mice suggesting that the olfactory receptor neurons play a key role in generating neural responses with respect to odors. So far, none of the chemical synapses have been known in signal transduction between olfactory receptor neurons, but the existence of gaseous second receptors or gap junctions or nitric oxide-mediated controls were evidenced as candidate for synchronizing neuronal firings (Breer et al., 1992; Levey et al., 1992; Ingi and Ronnett, 1995). Our observation of the significant reduction in the baseline delta power in ZnSO_4_–treated mice suggests the critical role of olfactory receptor neurons in genesis of delta oscillations.

Interestingly, we have observed that the baseline neuronal oscillations in the brain also changed as well. Recent reports suggest that mice olfactory deprived using ZnSO_4_ are more likely to develop cognitive and emotional impairments in chronic stress conditions (Chen et al., 2019) and have increased depressive and anxiolytic behaviors accompanied by a reduction of amygdalar corticotropin-releasing hormone (Ahn et al., 2018). To the best of our knowledge, the influence of the basal level dropping of the excitatory afferent sensory inputs on the resting-state activities of neurons or synaptic plasticity has not been reported. Nonetheless, some early sensory deprivation studies have reported an alteration in EEG alpha activities accompanied by hallucinatory experience (Hayashi et al., 1992), reflecting the possibility of critical influence of olfaction deprivation on the synaptic plasticity of neurons in the central nervous system.

## Conclusion

In conclusion, our study using the oddball paradigm showed that impairments in the peripheral regions of the olfactory system may be revealed by measuring neural oscillations during passive odor stimulation. To the best of our knowledge, it is the first time that an olfactory oddball paradigm is used in relation with local field potential recording, and even under urethane anesthesia, a different oscillatory pattern was recorded for the deviant odor, independently of the odor used. Still, this study has limitations in acquiring dissociable signals from urethane or respiration to dissect the bottom-up signal processing of odor from the systemic alterations. Simultaneous monitoring of physiological parameters such as breathing or blood pressure as well as investigation of dose-dependency could be explored in future experiments. Lastly, these neural oscillation signatures may be utilized in establishing diagnostic guidelines for olfactory disorder patients.

## Ethics Statement

All surgical and experimental procedures were followed by Korean Animal and Plant Quarantine Agency Publication No. 12512, partial amendment 2014, conforming to NIH guidelines (NIH Publication No. 86–23, revised 1985). All the procedures were approved by the Institutional Animal Care and Use Committee of the Korean Institute of Science and Technology (AP-2014L7002).

## Author Contributions

JC and J-HY conceptualized and designed the study. JK, JC, and J-HY wrote the manuscript. JK, J-GH, H-JC, and C-HK prepared the animals for surgery. JWK and OB built the experimental setup. JK and JWK acquired the data. JK, H-BH, and JC participated in data analysis and interpretation. All authors reviewed the manuscript.

## Conflict of Interest Statement

The authors declare that the research was conducted in the absence of any commercial or financial relationships that could be construed as a potential conflict of interest.

## Footnotes

1 https://mouse.brain-map.org
