# Supplementary material for: Neural Dynamics of Olfactory Perception: Low- and High-Frequency Modulations of Local Field Potential Spectra in Mice Revealed by an Oddball Stimulus
Source: Front Neurosci. 2019 May 28;13:478. doi: 10.3389/fnins.2019.00478 (PMC6546879; doi:10.3389/fnins.2019.00478)
Supplement: Supplementary file 1 [file Data_Sheet_1.PDF]

Supplementary Figures for

Manuscript # = 450020 (Frontiers in Neuroscience)

Analysis of odour-evoked neural oscillation patterns from both the peripheral and central nervous systems using an olfactory oddball paradigm

Jeungeun Kum[1,2], Jin Won Kim[3], Hyung-Ju Cho[3], Jong-Gyun Ha[3], Chang-Hoon Kim[3,4], Hio-Been Han[1,5], Jee Hyun Choi[1,2*] and Joo-Heon Yoon[3,4*],

Supplementary Figure S1. The power spectral density of baseline and the relative power during stimulation compared to baseline for the LFPs. Horizontal red lines indicate significant differences in the power between standard and deviant stimuli (Welch's *t*-test, p < 0.05).

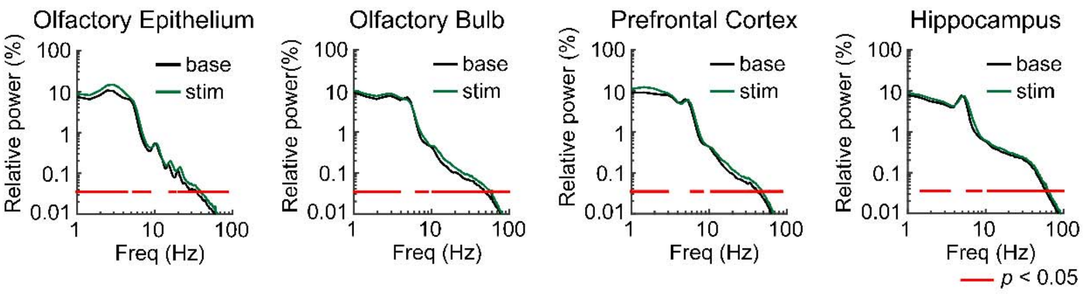

Supplementary Figure S2. The power as a function of time. The values were obtained by average over 70 trials and over 5 mice. (A) total power, (B) δ power, (C) θ power, (D) β power, (E) low γ power, and (F) high γ power.

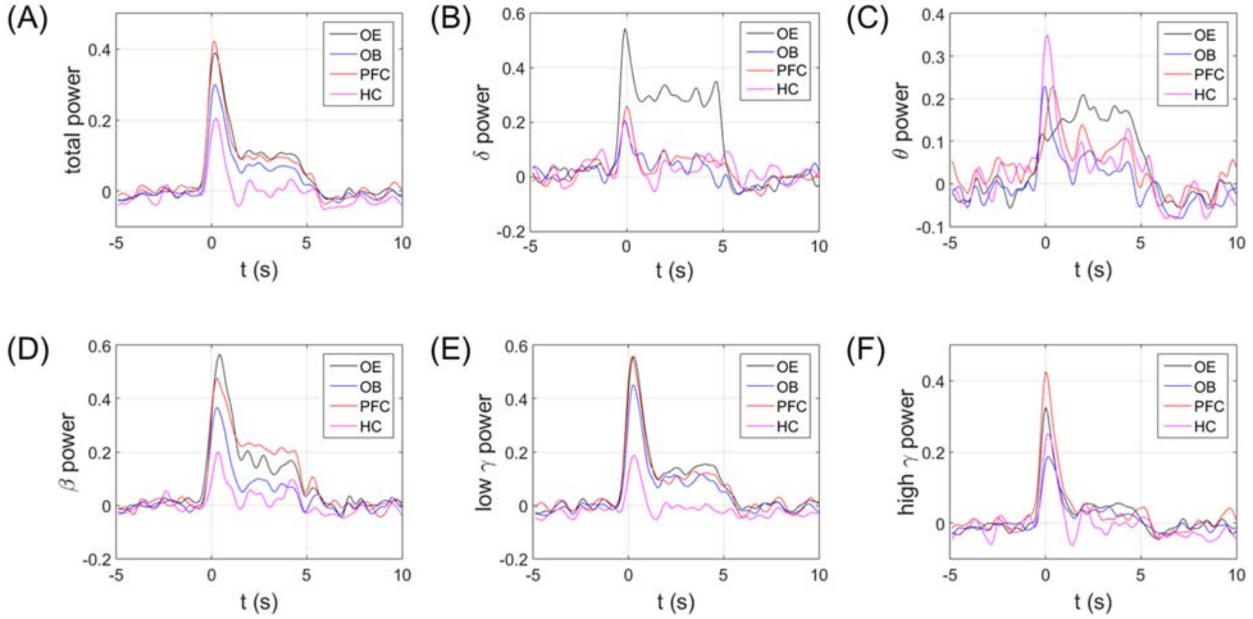

Supplementary Figure S3. The power changes across the trials displayed as scatter plots. The early 2 s and late 2s periods were separately analysed. Linear regression analysis showed that statistically significant decrease in δ, θ, and β powers of OE across the trials. In brain regions, θ and low γ powers of OB and low γ power of PFC significantly decreased as the trials continued. One-sided t-test was performed to test the negativity of the slope. (A) total power, (B) δ power, (C) θ power, (D) β power, (E) low γ power, and (F) high γ power.

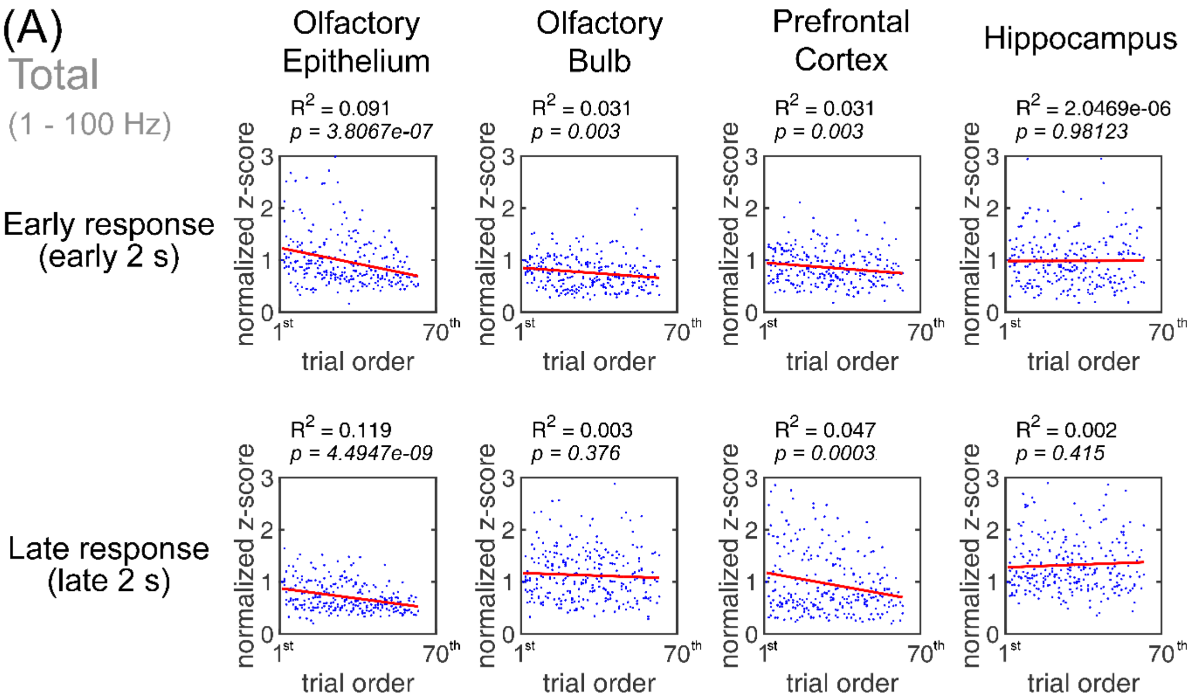

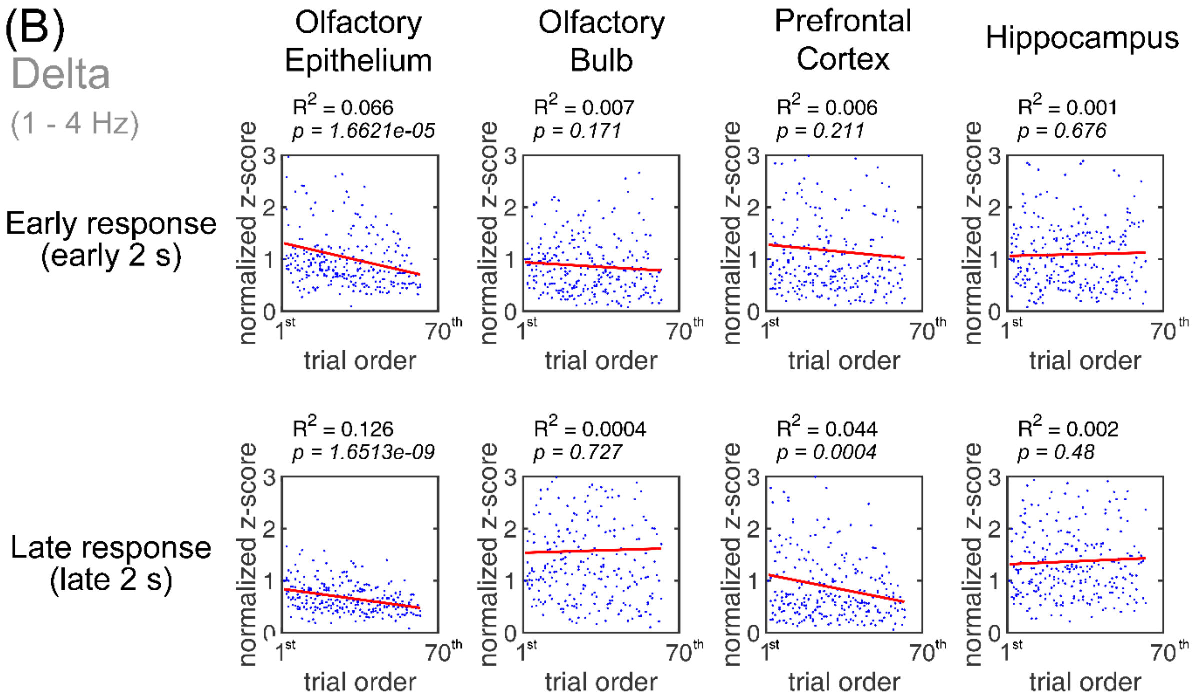
(B)
Delta
(1 - 4 Hz)
Olfactory Epithelium
Olfactory Bulb
Prefrontal Cortex
Hippocampus
Early response (early 2 s)
Late response (late 2 s)
R2 = 0.066
p = 1.6621e-05
R2 = 0.007
p = 0.171
R2 = 0.006
p = 0.211
R2 = 0.001
p = 0.676
R2 = 0.126
p = 1.6513e-09
R2 = 0.0004
p = 0.727
R2 = 0.044
p = 0.0004
R2 = 0.002
p = 0.48
normalized z-score
trial order
1st
70th

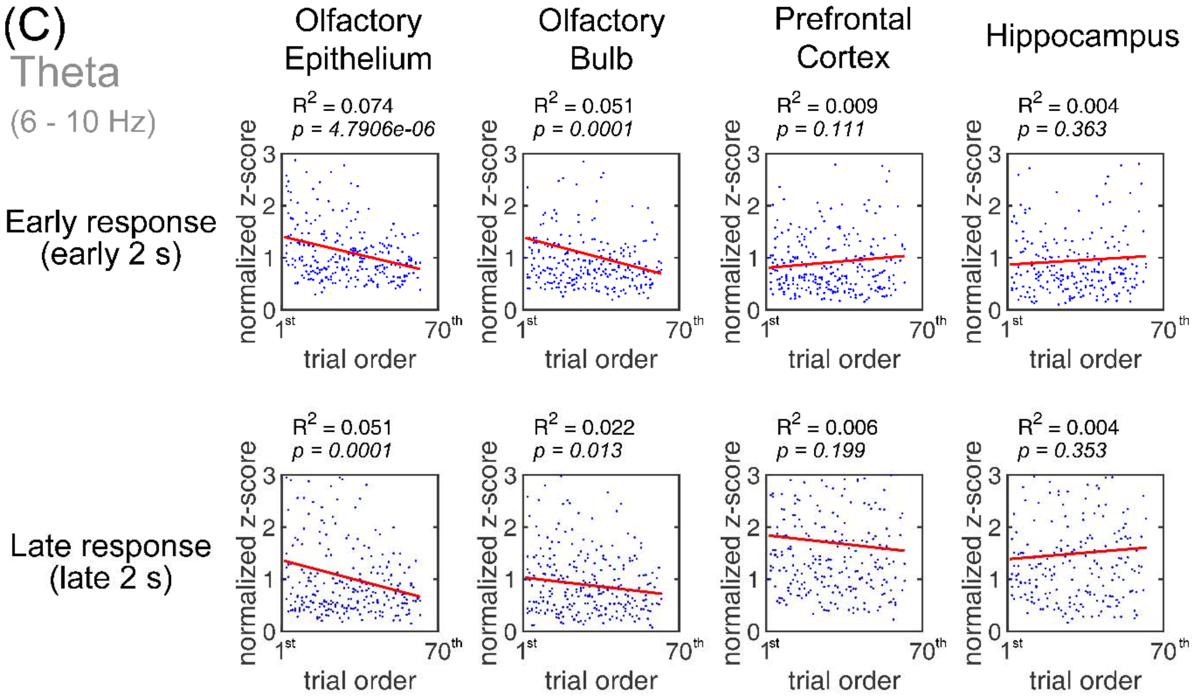
(C)
Theta
(6 - 10 Hz)
Olfactory Epithelium
Olfactory Bulb
Prefrontal Cortex
Hippocampus
Early response (early 2 s)
Late response (late 2 s)
R2 = 0.074
p = 4.7906e-06
R2 = 0.051
p = 0.0001
R2 = 0.009
p = 0.111
R2 = 0.004
p = 0.363
R2 = 0.051
p = 0.0001
R2 = 0.022
p = 0.013
R2 = 0.006
p = 0.199
R2 = 0.004
p = 0.353
normalized z-score
trial order
1st
70th

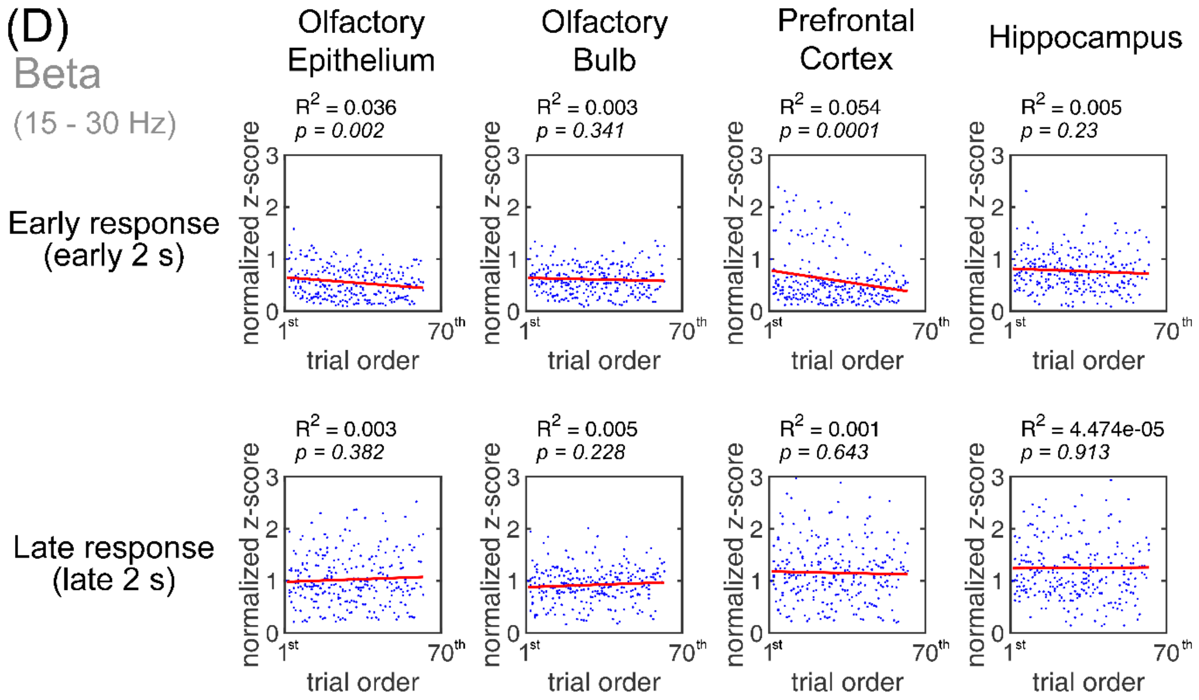
(D)
Beta
(15 - 30 Hz)
Olfactory Epithelium
Olfactory Bulb
Prefrontal Cortex
Hippocampus
Early response (early 2 s)
Late response (late 2 s)
R2 = 0.036
p = 0.002
R2 = 0.003
p = 0.341
R2 = 0.054
p = 0.0001
R2 = 0.005
p = 0.23
R2 = 0.003
p = 0.382
R2 = 0.005
p = 0.228
R2 = 0.001
p = 0.643
R2 = 4.474e-05
p = 0.913
normalized z-score
trial order
1st
70th

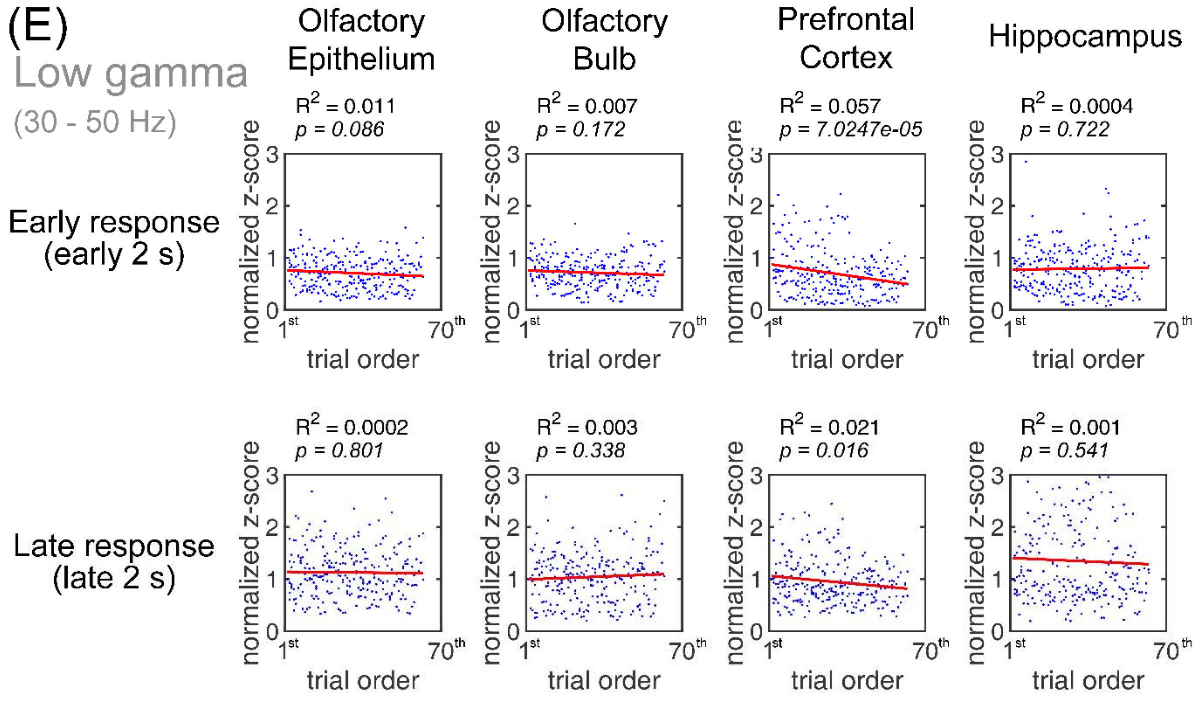
(E)
Low gamma
(30 - 50 Hz)
Olfactory Epithelium
Olfactory Bulb
Prefrontal Cortex
Hippocampus
Early response (early 2 s)
Late response (late 2 s)
R2 = 0.011
p = 0.086
R2 = 0.007
p = 0.172
R2 = 0.057
p = 7.0247e-05
R2 = 0.0004
p = 0.722
R2 = 0.0002
p = 0.801
R2 = 0.003
p = 0.338
R2 = 0.021
p = 0.016
R2 = 0.001
p = 0.541
normalized z-score
trial order
1st
70th

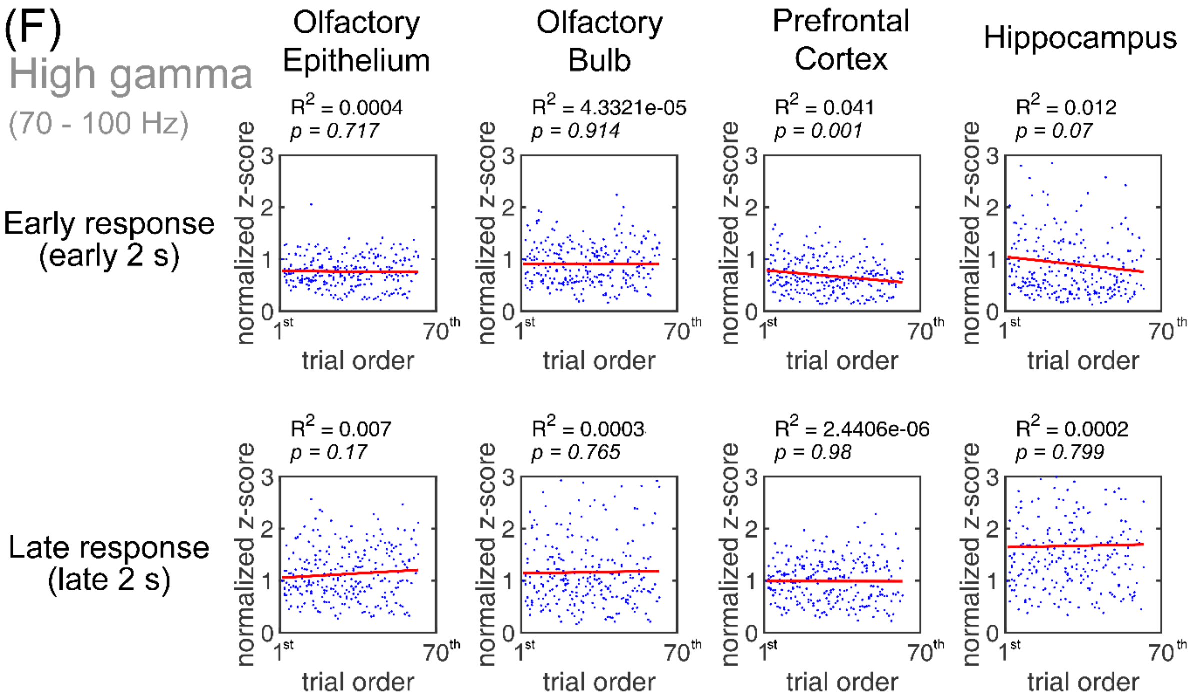
(F)
High gamma
(70 - 100 Hz)
Olfactory Epithelium
Olfactory Bulb
Prefrontal Cortex
Hippocampus
Early response (early 2 s)
Late response (late 2 s)
R2 = 0.0004
p = 0.717
R2 = 4.3321e-05
p = 0.914
R2 = 0.041
p = 0.001
R2 = 0.012
p = 0.07
R2 = 0.007
p = 0.17
R2 = 0.0003
p = 0.765
R2 = 2.4406e-06
p = 0.98
R2 = 0.0002
p = 0.799
normalized z-score
trial order
1st
70th

Supplementary Figure S4. The averaged z-score power spectrograms of control mice group which methyl salicylate was used as standard odour while ethyl acetate was used as deviant odour. To obtain a z-score spectrogram, the mean of the baseline power was subtracted from the spectrogram and then divided by the mean of the baseline power. The z-score spectrograms from all standard trials (methyl salicylate) and deviant trials (ethyl acetate) were averaged separately and then displayed. The bar graphs underneath the spectrograms indicate the statistical significance of baseline vs. stimulus (A, B) or standard vs. deviant (C) at each frequency band (paired *t*-test). Statistical tests were performed for the δ (1–4 Hz), θ (6–10 Hz), β (15–30 Hz), low γ (30–50 Hz), and high γ (70–100 Hz) frequency bands.

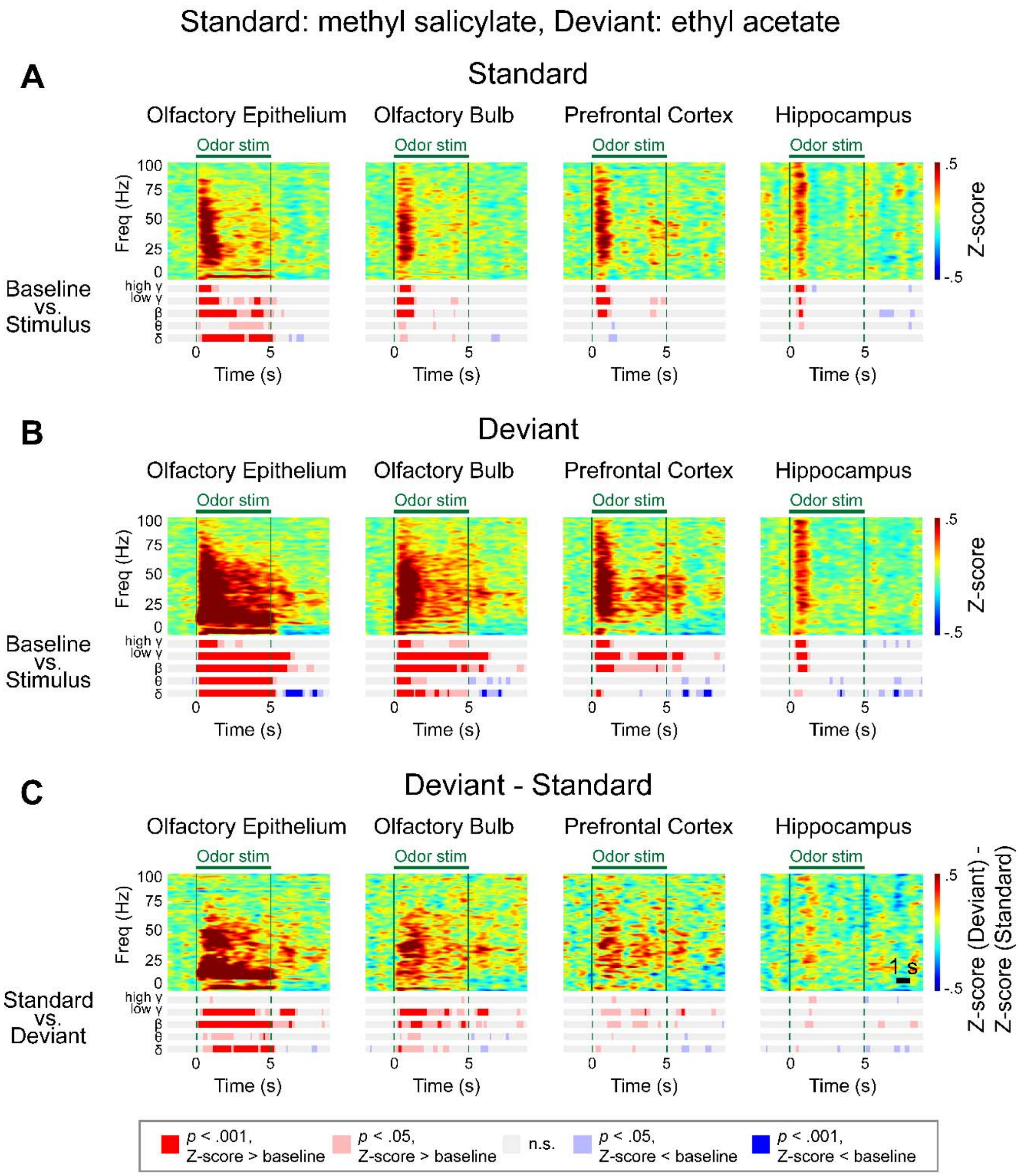
Standard: methyl salicylate, Deviant: ethyl acetate
A
Standard
B
Deviant
C
Deviant - Standard
Olfactory Epithelium
Olfactory Bulb
Prefrontal Cortex
Hippocampus
Odor stim
Freq (Hz)
100
75
50
25
0
high γ
low γ
β
θ
δ
Baseline vs. Stimulus
Standard vs. Deviant
Time (s)
0
5
.5
-.5
Z-score
Z-score (Deviant) - Z-score (Standard)
1 s
p < .001, Z-score > baseline
p < .05, Z-score > baseline
n.s.
p < .05, Z-score < baseline
p < .001, Z-score < baseline

Supplementary Figure S5. The averaged z-score power spectrograms of control mice group which ethyl acetate was used as standard odour while methyl salicylate was used as deviant odour. To obtain a z-score spectrogram, the mean of the baseline power was subtracted from the spectrogram and then divided by the mean of the baseline power. The z-score spectrograms from all standard trials (ethyl acetate) and deviant trials (methyl salicylate) were averaged separately and then displayed. The bar graphs underneath the spectrograms indicate the statistical significance of baseline vs. stimulus (A, B) or standard vs. deviant (C) at each frequency band (paired *t*-test). Statistical tests were performed for the δ (1–4 Hz), θ (6–10 Hz), β (15–30 Hz), low γ (30–50 Hz), and high γ (70 – 100 Hz) frequency bands.

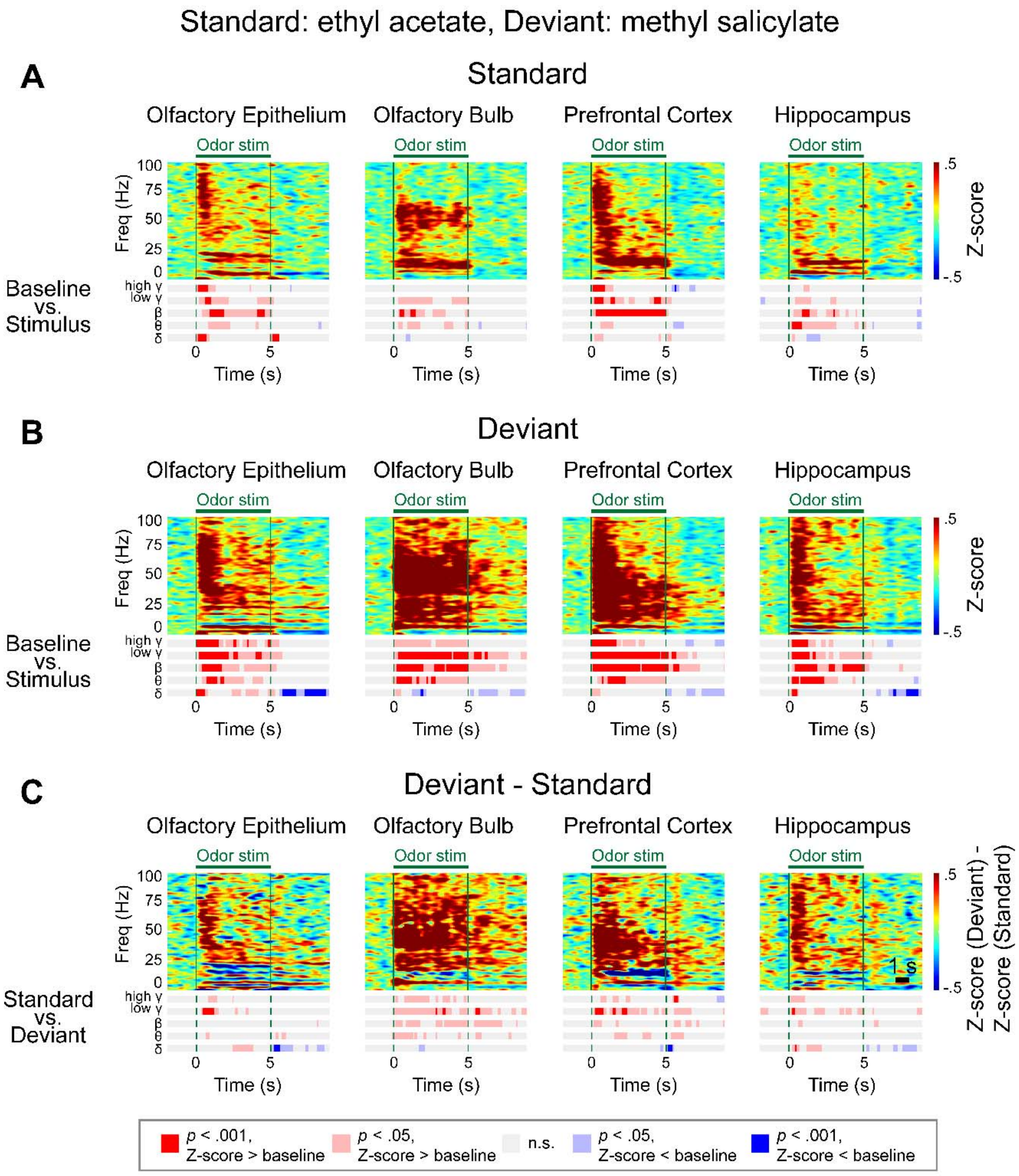
Standard: ethyl acetate, Deviant: methyl salicylate
A
Standard
Olfactory Epithelium
Olfactory Bulb
Prefrontal Cortex
Hippocampus
Odor stim
Freq (Hz)
100
75
50
25
0
Z-score
.5
-.5
Baseline vs. Stimulus
high γ
low γ
β
θ
δ
0
5
Time (s)
B
Deviant
C
Deviant - Standard
Standard vs. Deviant
1 s
Z-score (Deviant) - Z-score (Standard)
p < .001, Z-score > baseline
p < .05, Z-score > baseline
n.s.
p < .05, Z-score < baseline
p < .001, Z-score < baseline

1

2

3

Supplementary Figure S6. The averaged z-score power spectrogram of all control mice. To obtain a z-score spectrogram, the mean of the baseline power was subtracted from the spectrogram and then divided by the mean of the baseline power. The z-score spectrograms from all standard trials and deviant trials were averaged separately and then displayed. The bar graphs underneath the spectrograms indicate the statistical significance of baseline vs. stimulus (A, B) or standard vs. deviant (C) at each frequency band (paired *t*-test). Statistical tests were performed for the δ (1–4 Hz), θ (6–10 Hz), β (15–30 Hz), low γ (30–50 Hz), and high γ (70–100 Hz) frequency bands.

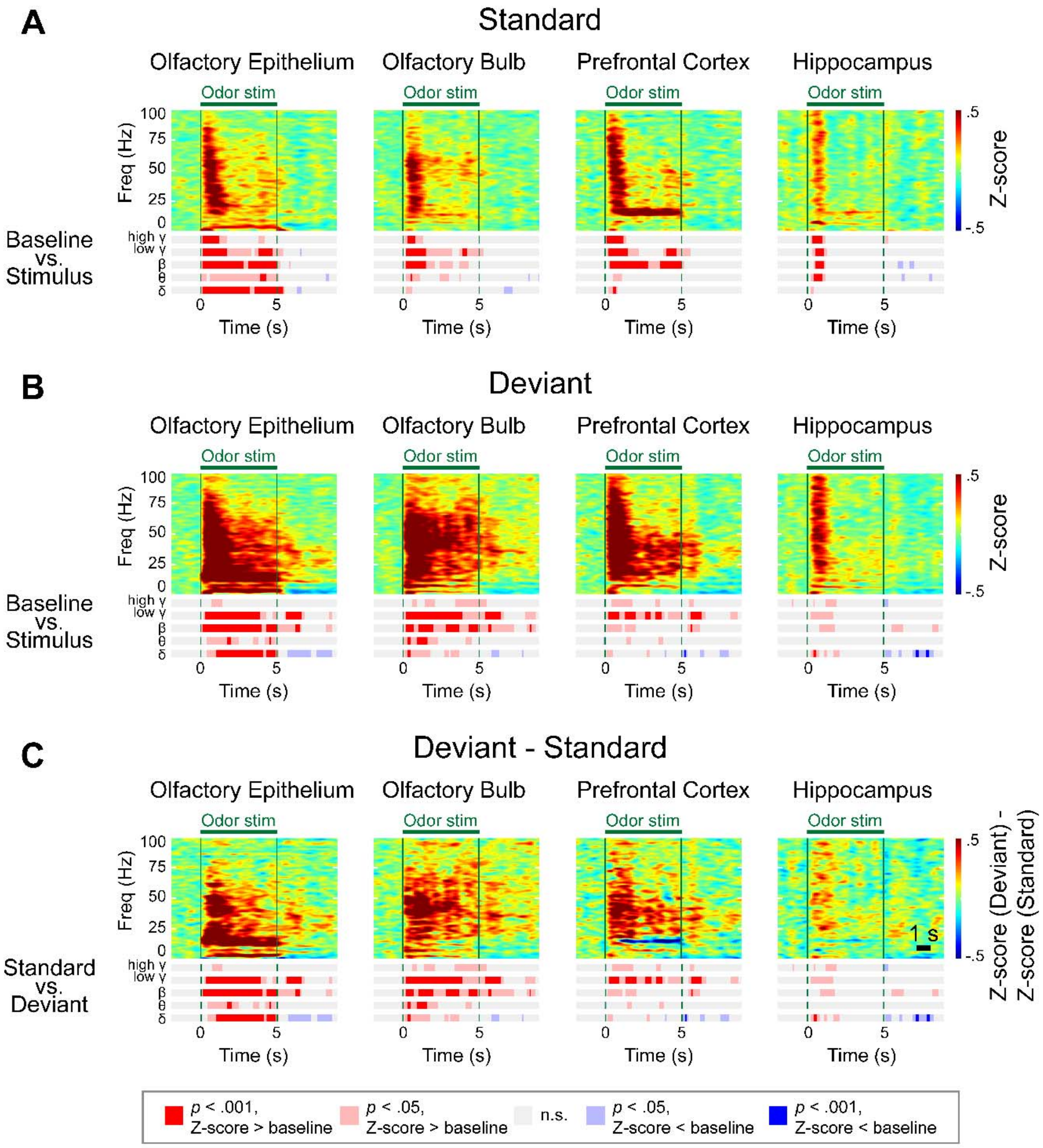
A
Standard
Olfactory Epithelium
Olfactory Bulb
Prefrontal Cortex
Hippocampus
Odor stim
Freq (Hz)
Z-score
Baseline vs. Stimulus
high γ
low γ
β
θ
δ
Time (s)
B
Deviant
C
Deviant - Standard
Standard vs. Deviant
1 s
Z-score (Deviant) - Z-score (Standard)
p < .001, Z-score > baseline
p < .05, Z-score > baseline
n.s.
p < .05, Z-score < baseline
p < .001, Z-score < baseline

Supplementary Figure S7. The averaged z-score powers of control and $ZnSO_4$-treated groups during 5-sec pre-stimulus period averaged over mice and over trials. Significantly different groups were mark by *** for p<0.001 (Wilcoxon signed-rank test).

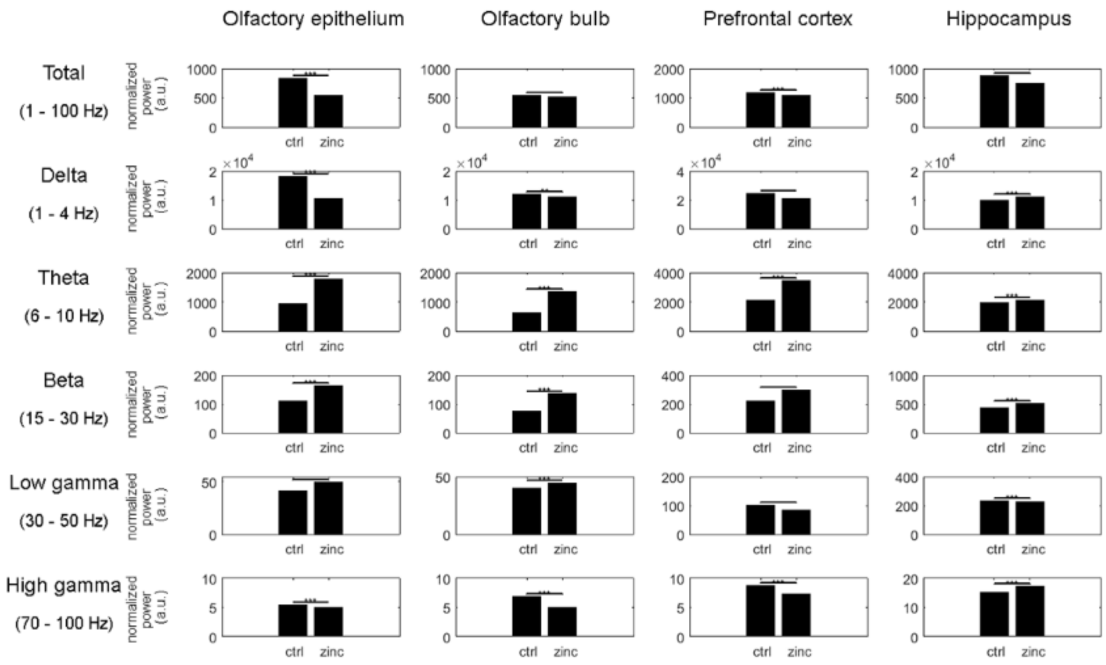

Supplementary Figure S8. The averaged z-score power spectrograms of $ZnSO_4$-treated mice group which methyl salicylate was used as standard odour while ethyl acetate was used as deviant odour. To obtain a z-score spectrogram, the mean of the baseline power was subtracted from the spectrogram and then divided by the mean of the baseline power. The z-score spectrograms from all standard trials (methyl salicylate) and deviant trials (ethyl acetate) were averaged separately and then displayed. The bar graphs underneath the spectrograms indicate the statistical significance of baseline vs. stimulus (A, B) or standard vs. deviant (C) at each frequency band (paired *t*-test). Statistical tests were performed for the δ (1–4 Hz), θ (6–10 Hz), β (15–30 Hz), low γ (30–50 Hz), and high γ (70–100 Hz) frequency bands.

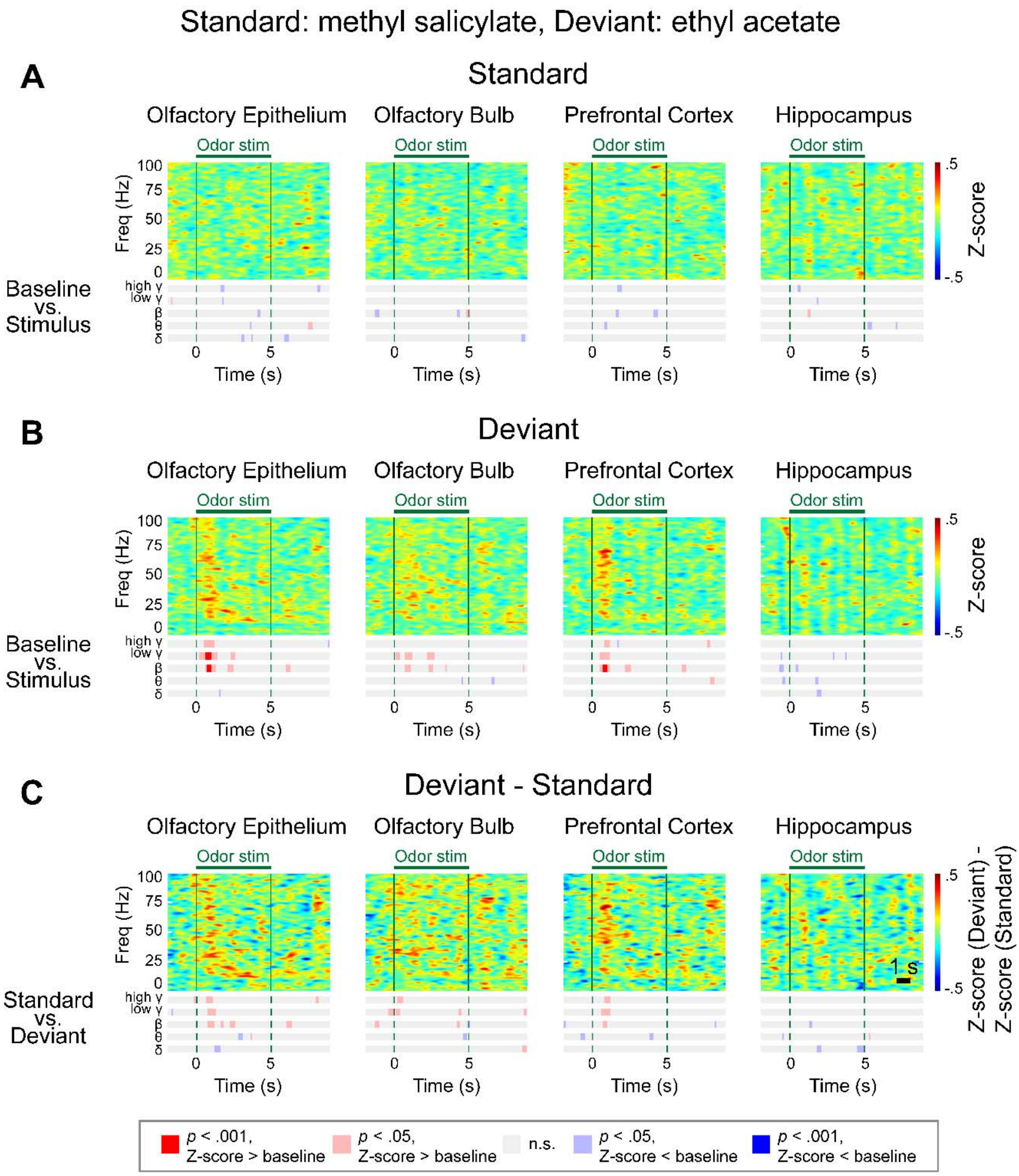
Standard: methyl salicylate, Deviant: ethyl acetate
A
Standard
B
Deviant
C
Deviant - Standard
Olfactory Epithelium
Olfactory Bulb
Prefrontal Cortex
Hippocampus
Odor stim
Freq (Hz)
Time (s)
Z-score
Baseline vs. Stimulus
Standard vs. Deviant
Z-score (Deviant) - Z-score (Standard)
high γ
low γ
β
θ
δ
1 s
p < .001, Z-score > baseline
p < .05, Z-score > baseline
n.s.
p < .05, Z-score < baseline
p < .001, Z-score < baseline

Supplementary Figure S9. The averaged z-score power spectrograms of $ZnSO_4$-treated mice group which ethyl acetate was used as standard odour while methyl salicylate was used as deviant odour. To obtain a z-score spectrogram, the mean of the baseline power was subtracted from the spectrogram and then divided by the mean of the baseline power. The z-score spectrograms from all standard trials (ethyl acetate) and deviant trials (methyl salicylate) were averaged separately and then displayed. The bar graphs underneath the spectrograms indicate the statistical significance of baseline vs. stimulus (A, B) or standard vs. deviant (C) at each frequency band (paired *t*-test). Statistical tests were performed for the δ (1–4 Hz), θ (6–10 Hz), β (15–30 Hz), low γ (30–50 Hz), and high γ (70–100 Hz) frequency bands.

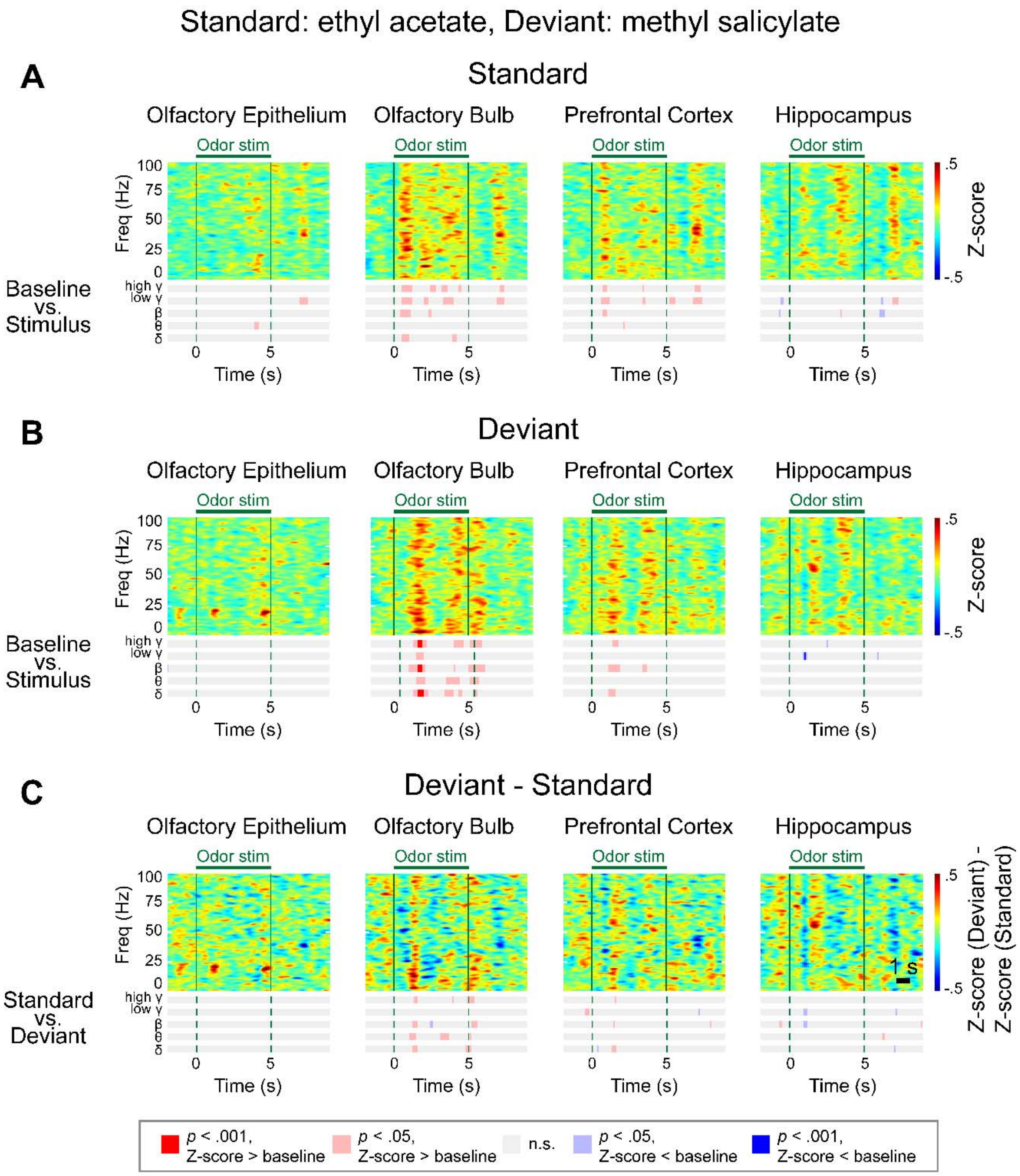

Standard: ethyl acetate, Deviant: methyl salicylate
A
Standard
B
Deviant
C
Deviant - Standard
Olfactory Epithelium
Olfactory Bulb
Prefrontal Cortex
Hippocampus
Odor stim
Freq (Hz)
100
75
50
25
0
Baseline vs. Stimulus
Standard vs. Deviant
high γ
low γ
β
θ
δ
Time (s)
0
5
Z-score
.5
-.5
Z-score (Deviant) - Z-score (Standard)
1 s
p < .001, Z-score > baseline
p < .05, Z-score > baseline
n.s.
p < .05, Z-score < baseline
p < .001, Z-score < baseline

Supplementary Figure S10. The averaged z-score power spectrogram of all $ZnSO_4$-treated mice. To obtain a z-score spectrogram, the mean of the baseline power was subtracted from the spectrogram and then divided by the mean of the baseline power. The z-score spectrograms from all standard trials and deviant trials were averaged separately and then displayed. The bar graphs underneath the spectrograms indicate the statistical significance of baseline vs. stimulus (A, B) or standard vs. deviant (C) at each frequency band (paired *t*-test). Statistical tests were performed for the δ (1–4 Hz), θ (6–10 Hz), β (15–30 Hz), low γ (30–50 Hz), and high γ (70–100 Hz) frequency bands.

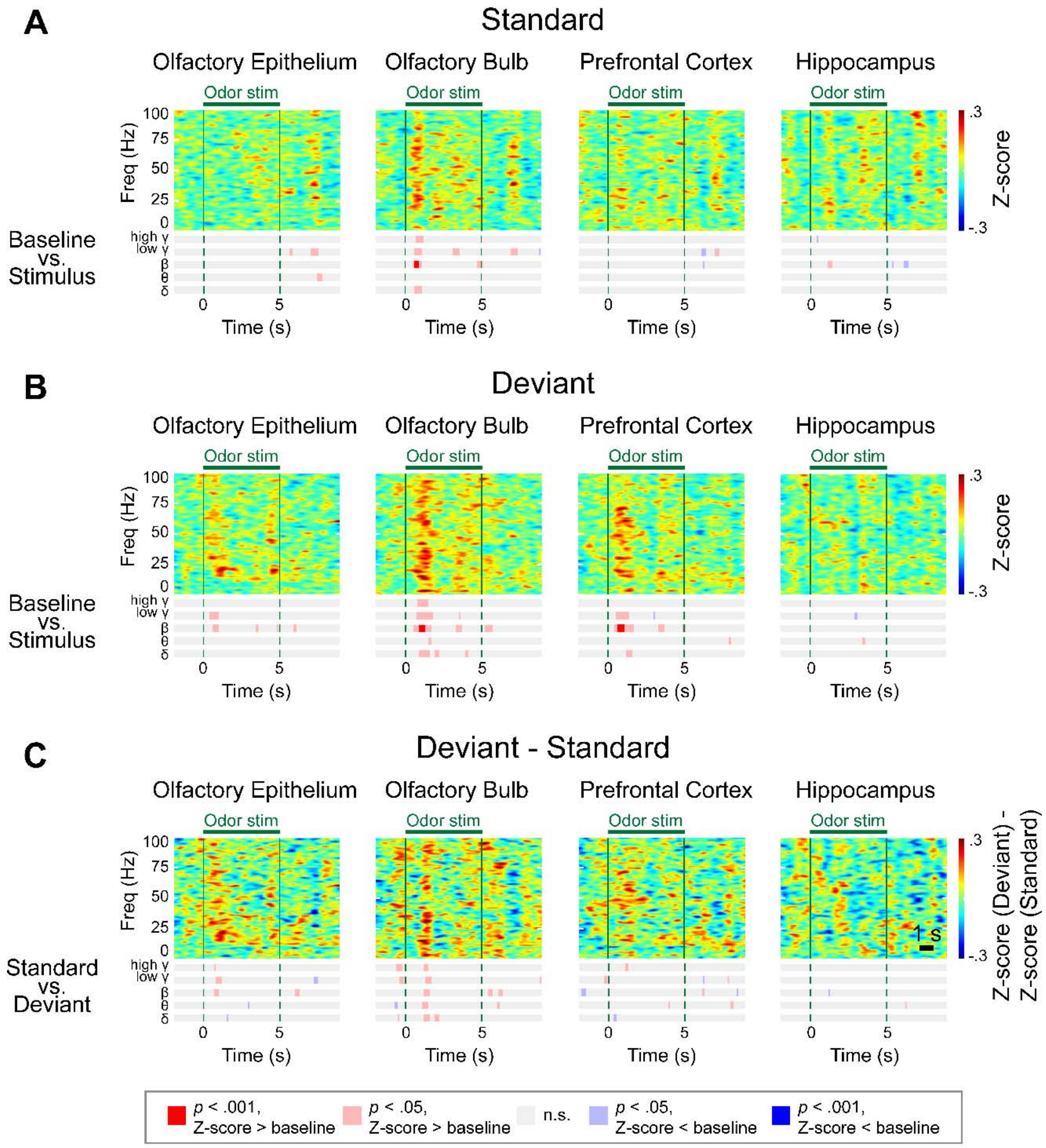
A
Standard
Olfactory Epithelium
Olfactory Bulb
Prefrontal Cortex
Hippocampus
Odor stim
Freq (Hz)
Z-score
Baseline vs. Stimulus
high γ
low γ
β
θ
δ
Time (s)
B
Deviant
C
Deviant - Standard
Standard vs. Deviant
Z-score (Deviant) - Z-score (Standard)
1 s
p < .001, Z-score > baseline
p < .05, Z-score > baseline
n.s.
p < .05, Z-score < baseline
p < .001, Z-score < baseline
